# Development of a Biodegradable Patch Based on Polysaccharides

**DOI:** 10.3390/polym17212908

**Published:** 2025-10-30

**Authors:** Gulzeinep Begimova, Aishat Kuldanova, Kenzhegul Smailova, Indira Kurmanbayeva

**Affiliations:** 1 Chemistry Department, S.D. Asfendiyarov Kazakh National Medical University, Tole by 94, Almaty 050012, Kazakhstan; kuldanova.aishat@kaznmu.kz (A.K.); kurmanbayeva.i@kaznmu.kz (I.K.); 2 Chemistry Department, Kazakh National Women’s Teacher Training University, Almaty 050000, Kazakhstan; smailova.k@kaznmu.kz; 3 Chemistry Department, Abai Kazakh National Pedagogical University, Almaty 050010, Kazakhstan

**Keywords:** polysaccharide hydrogel, gellan gum, chitosan, agar–agar, glutaraldehyde crosslinking, transdermal drug delivery, wound healing patch, methylene blue

## Abstract

Transdermal hydrogel films were fabricated from gellan gum, chitosan, and agar–agar, employing glutaraldehyde as a covalent crosslinker. The obtained formulation exhibited structural stability, pH-sensitive swelling, and high biocompatibility without the participation of metal ions. FTIR spectra showed the emergence of a characteristic imine (C=N) vibration near 1630 cm^−1^, confirming covalent network formation through Schiff-base reactions. SEM imaging revealed a homogeneous porous architecture (45–120 μm) that enhances moisture absorption and molecular diffusion. The swelling ratio reached 410 ± 12% at pH 9.18 and 275 ± 9% at pH 4.01, evidencing pronounced pH responsiveness. Mechanical strength measured 0.82 ± 0.03 MPa with elongation of 42 ± 2%, ensuring flexibility for skin application. The temperature-controlled release of methylene blue achieved 78 ± 4% at 40 °C after 24 h, consistent with diffusion-limited transport. This gellan–chitosan–agar hydrogel network crosslinked with glutaraldehyde represents a stable, pH-responsive, and biocompatible platform suitable for wound care and transdermal drug delivery.

## 1. Introduction

Transdermal drug delivery systems are polymer-based compositions in the form of patches that are applied to the skin to provide controlled and predetermined drug release for systemic action. This method of administration is effective for the treatment of a number of clinical conditions, enabling the active substance to bypass the gastrointestinal tract and penetrate through the skin barrier directly into the bloodstream. In recent years, numerous types of patches with different mechanisms of action, properties, and excipients have been developed and tested, each offering distinct advantages and limitations [1].

Polysaccharides such as gellan gum, chitosan, and agar–agar were selected as the polymeric framework in this study. Among these, gellan gum has attracted particular attention due to its excellent physicochemical, mechanical, and functional properties, which offer broad opportunities for biomedical applications. It is non-toxic, easily forms gels, exhibits mucoadhesiveness and demonstrates high stability, biodegradability, and biocompatibility [2,3]. By combining gellan gum with other natural or synthetic polymers through cross-linking agents, its mechanical properties can be tuned, allowing the fabrication of scaffolds or dressings that are easy to apply to wounds [4,5]. However, a major limitation of gellan-based dressings in infected wound treatment is their lack of intrinsic antibacterial activity against both Gram-positive and Gram-negative bacteria. This limitation is the main rationale for incorporating additional copolymers or active agents into the polymer composite [6,7,8,9,10,11]. Furthermore, disadvantages such as the high melting point (90 °C), elevated gelation temperature, and reduced mechanical stability of gellan-based systems—caused by the exchange of divalent cations with monovalent ones over time—must also be addressed [12].

Chitosan, a natural cationic polysaccharide, has attracted significant attention in the development of biomedical devices due to its unique physicochemical and biological properties. Its positive charge distinguishes it from most other polysaccharides and enables interactions with negatively charged biomolecules, providing antimicrobial activity, hemostatic potential, and enhanced cellular responses [13]. Various chitosan-based systems have been reported, such as transdermal patches of isosorbide dinitrate prepared from chitosan and polyvinyl alcohol (1:9), and plasticized chitosan–starch patches (4:1) [14]. However, these formulations still face limitations, including relatively low mechanical strength, susceptibility to enzymatic degradation, and reduced stability under highly acidic or alkaline conditions.

Despite certain limitations, chitosan remains one of the most promising natural polymers for wound dressing materials because of its biocompatibility, biodegradability, and versatile hydrogel-forming ability. Hydrogels derived from chitosan can maintain a moist environment, efficiently absorb wound exudates, and form a semi-permeable barrier that protects the injured tissue from microbial contamination, thereby accelerating the healing process [15,16,17]. From a biological standpoint, chitosan enhances platelet and erythrocyte aggregation, supporting hemostasis in the initial wound phase, while its intrinsic antibacterial activity helps suppress infection and inflammation [18]. In later stages, chitosan stimulates fibroblast proliferation, granulation tissue formation, and extracellular matrix deposition, facilitating tissue remodeling and closure of the wound [19,20].

The functional performance of chitosan hydrogels largely depends on the chosen crosslinking strategy. Two main approaches are widely applied: chemical crosslinking and radiation-induced crosslinking. Chemical crosslinking involves initiator-induced polymerization or UV irradiation in the presence of photoinitiators, forming covalent networks with enhanced mechanical stability. For example, highly porous chitosan hydrogels have been fabricated using ethylene glycol and foaming methods, while UV-induced crosslinking with polyethylene glycol diacrylate and Aloe vera extract has also been reported [10,21]. In contrast, radiation-induced methods, such as γ-irradiation, enable the synthesis of chitosan-based composite hydrogels (e.g., CS/Gel/PVA), yielding materials with favorable mechanical properties, pH sensitivity, swelling behavior, and water retention [22].

Physically crosslinked chitosan hydrogels are generally safer, as they rely on biocompatible polymers without additional chemical agents. Nevertheless, they often exhibit insufficient stability, weak tissue adhesion, and limited mechanical strength. Chemically crosslinked hydrogels, while more robust and adaptable to different wound types, require extensive biocompatibility evaluation and sophisticated processing techniques, which may increase production costs and raise environmental concerns. Therefore, the rational design of chitosan-based wound dressings requires balancing biological safety with functional performance, selecting the optimal crosslinking approach to meet specific clinical needs [23].

Agar, a natural polysaccharide extracted primarily from red algae (Rhodophyceae), has gained attention as a versatile biopolymer in the development of biomedical composites. Its structure is mainly composed of agarose and agaropectin, which confer excellent gelling ability, high water retention, and biocompatibility [24,25]. These properties make agar particularly attractive as an additive or matrix component in hydrogel-based wound dressings and transdermal patches.

In composite systems, agar improves the mechanical strength, moisture retention, and swelling behavior of hydrogels, thereby enhancing their performance as wound dressings. For instance, agar has been blended with chitosan to form hydrogel patches with improved porosity, controlled release of therapeutic agents, and accelerated wound healing capacity [26]. Such chitosan–agar composites have demonstrated effective exudate absorption, antibacterial activity, and cytocompatibility, making them suitable candidates for biomedical use.

Moreover, agar contributes to the structural stability of hydrogel matrices under physiological conditions. Its thermo-reversible gelation behavior allows for facile processing and tunable mechanical properties, which can be advantageous in fabricating patches or films for controlled drug delivery [27]. In addition, agar-based materials are generally recognized as safe (GRAS), further supporting their application in biomedicine.

Nevertheless, agar-containing composites may exhibit brittleness and limited elasticity, which restrict their direct application. To overcome these limitations, agar is often combined with other natural or synthetic polymers (e.g., chitosan, gelatin, polyvinyl alcohol) to achieve synergistic effects in terms of flexibility, degradability, and bioactivity [28]. Such hybrid systems have been shown to enhance wound healing outcomes by maintaining a moist wound environment, reducing infection risk, and enabling the controlled release of bioactive molecules.

Taken together, agar serves as a valuable functional additive in polymeric composites for biomedical applications, particularly in wound dressings and patches. Its unique physicochemical properties, combined with complementary polymers, offer opportunities for the design of multifunctional and clinically applicable biomaterials. Therefore, the aim of this study was to develop and characterize composite patches based on gellan gum, chitosan, and agar–agar, designed for potential use as transdermal drug delivery systems. The work focused on assessing the influence of polymer composition and crosslinking conditions on the structural integrity, swelling behavior, and drug release properties of the obtained patches under physiologically relevant conditions.

## 2. Materials and Methods

### 2.1. Chemicals and Reagents for Synthesis

Agar, gellan gum, and chitosan were used as polysaccharide components in this study. Agar was employed as microbiological grade powder (Sigma-Aldrich, Cat. No. 01916, Agar powder, CAS 9002-18-0, Darmstadt, Germany). Gellan gum was used as Gelrite^®^ (Sigma-Aldrich, Cat. No. G1910, CAS 71010-52-1, Darmstadt, Germany), a highly purified polysaccharide commonly applied in biotechnological media. Chitosan was used in its low molecular weight form (Sigma-Aldrich, Cat. No. 448869, low molecular weight chitosan, CAS 9012-76-4, Darmstadt, Germany) with an approximate molecular weight of 150–250 kDa and a degree of deacetylation (DDA) of about 75–85%, according to the manufacturer’s technical information. All reagents were of laboratory grade and used without further purification.

### 2.2. Preparation of Polymer Patches

#### 2.2.1. Gellan–Chitosan Patches (Samples G1 and G2)

Gellan gum (1 g) was dissolved in 50 mL of distilled water under magnetic stirring at 70 °C until a clear and homogeneous solution was obtained. Separately, chitosan was dispersed in distilled water to prepare colloidal solutions containing 0.25 g in 25 mL (for sample G1) and 0.5 g in 50 mL (for sample G2). The colloidal solution of chitosan was gradually added to the gellan gum solutions under continuous stirring to ensure complete homogenization. Subsequently, 2.5 mL (G1) or 5 mL (G2) of 25% aqueous glutaraldehyde was introduced as a crosslinking agent. The resulting viscous and transparent mixtures were cast into Petri dishes and left to stand for 24 h to complete initial gelation. The formed hydrogels were then subjected to additional ionic cross-linking in 0.1 M NaCl and 0.05 M CaCl_2_ solutions for 30 min to enhance their structural stability. Once ready, all samples were thoroughly washed in distilled water to remove unbound reagent residues.

#### 2.2.2. Agar–Chitosan Patches (Samples A3 and A4)

Agar–agar (1 g) was dissolved in 50 mL of hot distilled water under constant stirring until a clear and homogeneous solution was obtained. Separately, a colloidal solution of chitosan was prepared by dissolving 0.25 g (for sample A3) or 0.5 g (for sample A4) of chitosan in 25 mL or 50 mL of distilled water, respectively. The chitosan solutions were gradually added to the agar solutions under stirring to ensure homogeneity. Subsequently, 2.5 mL (A3) or 5 mL (A4) of glutaraldehyde was introduced as a cross-linking agent. The resulting mixtures were cast into Petri dishes for gel formation. No additional cross-linking with inorganic salts was required, as the obtained hydrogels exhibited sufficient structural stability and integrity. Once ready, all samples were thoroughly washed in distilled water to remove unbound reagent residues.

#### 2.2.3. Combined Gellan–Agar–Chitosan Patches (Samples C5 and C6)

For sample C5, 0.5 g of gellan gum was dissolved in 35 mL of distilled water under magnetic stirring until a clear solution was obtained. Separately, 0.25 g of chitosan was dispersed in 25 mL of distilled water, and 0.5 g of agar–agar was dissolved in 25 mL of hot distilled water under stirring. The chitosan and agar solutions were then gradually added to the gellan solution under continuous stirring to achieve homogeneity. Subsequently, 2.5 mL of 25% aqueous glutaraldehyde was introduced as a cross-linking agent.

For sample C6, 0.5 g of gellan gum was dissolved in 35 mL of distilled water under stirring, followed by the preparation of chitosan and agar solutions containing 0.125 g of chitosan in 15 mL of distilled water and 0.5 g of agar–agar in 25 mL of hot distilled water, respectively. The resulting solutions were combined under stirring, and 2.5 mL of 25% aqueous glutaraldehyde was added. The obtained mixtures were cast into Petri dishes and allowed to stand for 24 h to complete gelation. No additional ionic cross-linking was required, as the hydrogels demonstrated sufficient mechanical integrity. Once ready, all samples were thoroughly washed in distilled water to remove unbound reagent residues.

#### 2.2.4. Incorporation of Methylene Blue (Model Compound Samples)

To evaluate the loading efficiency and release kinetics, an additional series of composite samples (G1–G2 and A3–A4) containing methylene blue was prepared. Approximately 0.01 g of methylene blue was introduced into the warm, still-fluid polymer mixture during synthesis, immediately before the addition of the crosslinking agent. Preliminary observations indicated that incorporating the dye at this stage ensured more uniform distribution within the matrix and reduced leaching during subsequent ionic treatment with 0.1 M NaCl and 0.05 M CaCl_2_ solutions. In contrast, post-synthesis addition of methylene blue, followed by crosslinking, resulted in partial dye loss into the ionic medium, thereby decreasing the overall loading efficiency. The samples containing the dye were denoted with the suffix (e.g., G1-GM, A3-AM) and were subsequently used for loading and release studies.

### 2.3. Patch Thickness

The thickness of the patches was determined using a digital caliper at multiple points (3–5 measurements), including the corners and the center of each sample. The mean thickness and standard deviation were calculated from these measurements [21].

### 2.4. Folding Resistance

The folding resistance of the patches was evaluated manually by repeatedly folding the same area until visible cracks or tears appeared. The resistance was expressed as the number of folds sustained before damage occurred [22,29].

### 2.5. Swelling Study

The swelling behavior of polymer-crosslinked hydrogel patches was investigated to assess the influence of pH on their swelling capacity under topical application conditions. These buffer values were selected as conventional reference points to probe differences in ionization of polysaccharide functional groups and their effect on swelling dynamics. Dried patches (*W_o_*) were immersed in 250 mL of each buffer at room temperature over 72 h. At predetermined time points, patches were removed, gently blotted to remove surface moisture, and reweighed (*W_t_*). The process was repeated until equilibrium swelling (constant weight) was reached [30,31].

The swelling ratio (*S*, %) was calculated as:

(1) $$S=\;\frac{{W_{t}-\;W}_{o\;}}{W_{o}}\;\times 100\%\;$$

*W_t_*—the mass of the sample after swelling during t,

*W_o_*—the initial mass of the dry sample.

### 2.6. Sol–Gel Analysis

Sol–gel analysis was performed to determine the content of uncrosslinked components in the topical patches. The patches were first dried in an oven at 40 °C and weighed to obtain their initial dry mass. The dried patches were then immersed in 100 mL of distilled water for one week with occasional stirring to remove the soluble fraction. After one week, the patches were removed, carefully spread onto labeled Petri dishes, and redried in an oven at 40 °C until a constant mass was achieved [32,33,34,35]. The *Sol* and *Gel* fractions were calculated using the following equations:

(2) $$Gel=\;\frac{m_{d}}{m_{s}}\;\times 100\%$$

(3) Sol=100−Gel%

where *m_d_*—dry weight; *m_s_*—wet weight.

### 2.7. UV–Vis Spectroscopy Analysis

The release of methylene blue from the hydrogel patches was evaluated using UV–Vis spectrophotometry (Agilent Cary 60 UV–Vis, Agilent Technologies, Santa Clara, CA, USA). Drug release studies were carried out in phosphate buffer solutions of different pH values to simulate physiological conditions. Samples were withdrawn at predetermined time intervals, and the absorbance of the solutions was recorded in 1 cm quartz cuvettes at the characteristic absorption maximum of methylene blue (λ_max_ ≈ 660 nm). Calibration curves constructed from standard methylene blue solutions were used to determine the drug concentration in the release medium.

In parallel, the pH of the release medium was monitored using a calibrated pH meter equipped with a glass electrode to assess possible medium changes during the release process. All experiments were performed in triplicate to ensure reproducibility, and the cumulative release of methylene blue was expressed as a percentage of the total drug load. The mechanical resistance of the crosslinked polysaccharides (patches) was performed using a texture analyzer (TA-3000, LabSol, Beijing, China).

### 2.8. FTIR Spectroscopy and SEM Analysis

Sample spectra were recorded using a Nicolet Apex FTIR spectrometer (Thermo Fisher Scientific, Waltham, MA, USA) equipped with a Smart iTX attachment (ZnSe crystal, KBr beamsplitter, DTGS KBr detector). Ten scans per sample were collected at 4 cm^−1^ resolution with an accumulation time of 14.4 s per scan. Spectra were processed using strong N-B apodization and Mertz phase correction. The optical aperture was 80.0, with sample gain set to 1.0. All spectra were averaged to improve the signal-to-noise ratio.

The surface morphology of the developed hydrogel patches was investigated using a high-resolution scanning electron microscope JSM-6390 (JEOL Ltd., Tokyo, Japan). The analyses were performed in high-vacuum mode with a secondary electron detector at an accelerating voltage of 15 kV.

## 3. Results and Discussion

Over the past two decades, transdermal drug delivery systems (TDDS) have been recognized as a reliable and versatile technology offering several advantages over conventional routes of administration. In contrast to oral or parenteral delivery, transdermal systems enable controlled and sustained release of therapeutic agents, improved patient compliance, and reduced fluctuations in plasma drug concentrations [36,37]. Furthermore, the therapeutic effect can be easily discontinued by simply removing the patch, which provides an additional level of safety and dosing flexibility [38]. These advantages have stimulated intensive research into the development of novel polymeric systems capable of overcoming the intrinsic limitations of the skin barrier [39].

Biodegradable transdermal patches were developed using a composite of natural polysaccharides—gellan gum, chitosan, and agar–agar—selected for their biocompatibility, biodegradability, and complementary structural and rheological characteristics. The composition of the prepared hydrogel patches is summarized in Table 1. Gellan gum forms transparent, mechanically robust gels through ordered double-helix junctions, agar–agar contributes elasticity and smooth surface morphology, while chitosan enhances mucoadhesion, bioactivity, and electrostatic interaction with anionic polysaccharides. The incorporation of glutaraldehyde and divalent ions as crosslinking agents further reinforced the cohesion and durability of the polymeric network, enabling the formation of flexible and stable films. This synergistic combination allows fine-tuning of the mechanical strength–flexibility balance, providing a promising platform for transdermal drug delivery applications.

Methylene blue was used as a model hydrophilic compound due to its well-defined physicochemical properties, strong coloration, and analytical convenience, which facilitate quantitative assessment of diffusion and release kinetics. Although methylene blue does not fully replicate the behavior of therapeutic molecules, it provides valuable insight into the swelling and release mechanisms governing the developed systems. Future studies will incorporate pharmacologically active compounds to validate the release performance and therapeutic potential of the proposed polysaccharide-based patches.

While the present study offers comprehensive physicochemical and mechanical characterization of gellan–agar–chitosan patches, several limitations are acknowledged. Cytocompatibility and biocompatibility evaluations, kinetic modeling (Higuchi, Korsmeyer–Peppas), and statistical analysis of inter-sample variations were not performed. Addressing these aspects in future work will provide deeper understanding of the diffusion and erosion-controlled mechanisms underlying drug release and confirm the biomedical safety of the developed hydrogels.

Overall, the study highlights the potential of gellan–agar–chitosan composites as promising, biodegradable, and structurally stable matrices for controlled transdermal drug delivery applications.

**Figure 1:**
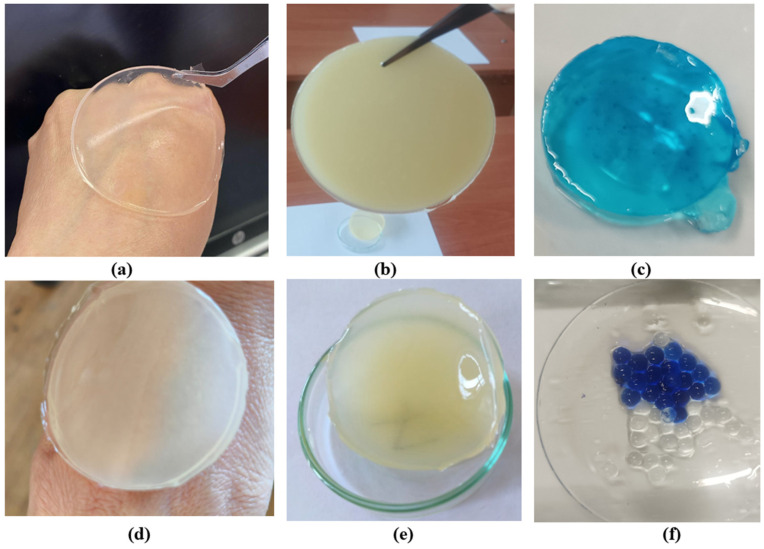
Representative hydrogel disks: gellan gum (**a**), agar (**b**), gellan with methylene blue (**c**), gellan–chitosan–agar composites (**d**,**e**), and gellan with methylene blue for release analysis (**f**).

Hydrogel disks were prepared from gellan gum, agar–agar, and chitosan, with gellan-based samples further subjected to ionic crosslinking using sodium and calcium salts following primary glutaraldehyde crosslinking. Introduction of multivalent cations increased gel stiffness and reduced aqueous solubility, resulting in stable disks that retained their shape after washing and drying, while agar–agar samples were not ionically treated. Figure 1 shows: (a) gellan gum disk; (b) agar disk; (c) gellan hydrogel containing methylene blue; (d,e) disks composed of gellan gum, chitosan, and agar; (f) gellan gum samples with methylene blue. Samples (c) and (f) were synthesized with the introduction of methylene blue to study the release of the target substance and its uniform distribution.

### 3.1. Weight Change and Thickness Evaluation

The average thickness of the fabricated patches was determined using three randomly selected specimens from each composition. Each sample was weighed individually, after which its thickness was measured with a digital caliper at five different points (the four edges and the central region). The mean thickness values ranged from 0.15 to 0.25 mm, with an average patch weight between 5.5 and 6.5 g (Table 2). The obtained results reflect the specific features of the preparation method: initially, the hot solution undergoes a transition from a thermally reversible random coil state to a more ordered double-helix conformation upon cooling, followed by the formation of a stable three-dimensional polymeric network through ion-induced cross-linking. Among the obtained materials, patches cross-linked with Ca^2+^ ions demonstrated superior structural stability and elasticity compared to those cross-linked with Na^+^ ions. This can be explained by the fact that monovalent sodium ions primarily reduce the electrostatic repulsion between carboxylate groups, whereas divalent calcium ions not only neutralize repulsion but also establish additional ionic bridges between polymer chains, thereby enhancing gel strength and integrity [40,41].

### 3.2. Bending Resistance

The mechanical flexibility of the hydrogel patches was evaluated in terms of bending resistance. All samples exhibited satisfactory performance, confirming that the incorporation of different polysaccharide concentrations resulted in elastic, non-brittle structures. Flexural resistance was assessed manually by repeatedly folding each specimen until visible cracks or rupture occurred. The number of folding cycles sustained before failure varied depending on the polymer composition. Notably, the A2 formulation demonstrated the highest resistance, withstanding up to 70 consecutive folds without structural failure, indicating its enhanced mechanical robustness compared to the other tested systems (Table 2).

### 3.3. Characterization of Transdermal Patches

In order to comprehensively evaluate transdermal patches, a series of physicochemical, mechanical, and biological tests are required. According to the recommendations of the European Medicines Agency (EMA) and the Committee for Medicinal Products for Human Use (CHMP), essential parameters include dissolution behavior, in vitro drug release, adhesive performance, and excipient compatibility [42,43,44]. In addition, a number of supporting analyses are necessary, such as the assessment of material interactions, patch thickness and weight uniformity, bending resistance, moisture content, moisture uptake, vapor permeability, drug loading, surface flatness, stability, swelling capacity, and potential for skin irritation. Together, these evaluations provide a comprehensive understanding of patch quality, stability, and suitability for therapeutic use. In this study, only a subset of these parameters was evaluated, focusing primarily on physicochemical and swelling properties.

### 3.4. Weight Loss Evaluation

The weight loss of hydrogel-based patches was examined over two distinct time intervals. The first stage focused on the initial dehydration process, where weight reduction was measured during the first 1.5 h of drying across all formulations. The second stage extended over several days, until the samples reached a constant weight, thereby reflecting their equilibrium water content. Following complete dehydration, the rehydration capacity (reversibility) of the hydrogel matrix was evaluated as an indicator of its structural stability and resilience.

### 3.5. Moisture Retention, Swelling Behavior, and Mechanical Properties of the Composite Patches

**Figure 2:**
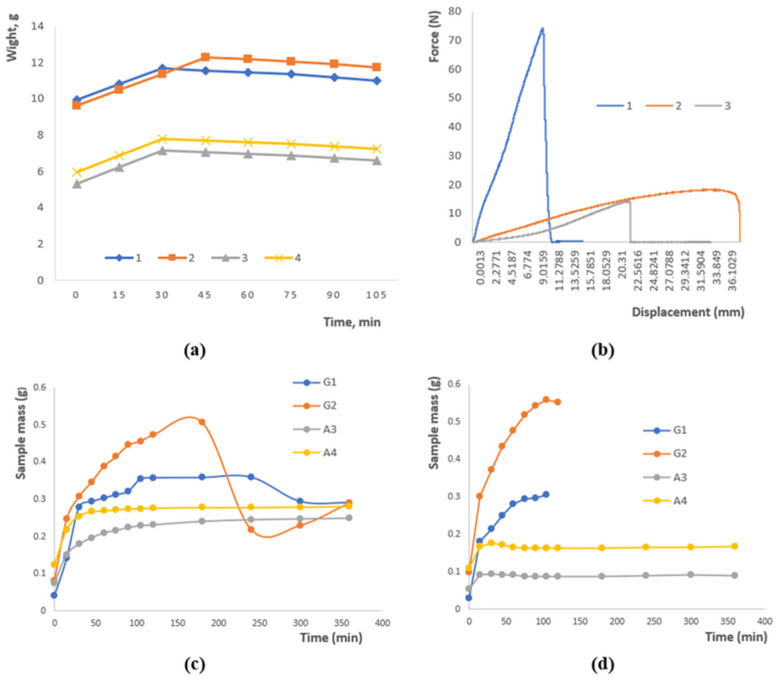
(**a**) Moisture loss of a sample as a function of time. (**b**) Research on the rupture of polymer patches. (**c**) Swelling at pH 4.01. (**d**) Swelling at pH 9.18. Data are presented as mean ± SD (*n* = 3); variations ≤ 5%; error bars omitted for clarity.

Figure 2a illustrates the variation in sample mass over time as a result of moisture loss. An initial rise in mass was observed for all samples within the first 30–45 min, which can be attributed to the hydration of the polymer network. After reaching a peak, the mass gradually approached equilibrium. Samples 1 and 2 maintained the highest mass values (~11–12 g), reflecting superior water-holding ability, whereas samples 3 and 4 exhibited lower mass (~6–8 g), likely due to differences in polysaccharide composition and crosslinking density. These findings demonstrate that both formulation and crosslinking degree significantly affect the water retention behavior of hydrogels.

The graph in Figure 2b presents the mechanical response of the polymer patches under applied stress. Sample 1 exhibits a rapid rise in force up to approximately 75 units, followed by an abrupt rupture, characteristic of brittle failure. In contrast, Samples 2 and 3 display a more gradual increase in load, reaching around 20 and 15 units, respectively, which reflects a ductile nature and higher deformation tolerance. These observations indicate that both the formulation and crosslinking degree govern the mechanical performance of the patches, with the combination of gellan, agar, and chitosan ensuring an effective balance between strength and flexibility.

Figure 2c depicts the swelling behavior of the samples under acidic conditions. Samples G2 and G1 exhibit the highest swelling capacity, reaching approximately 0.5 g for G2, whereas A3 and A4 remain comparatively stable at around 0.2–0.25 g. During the initial stage (0–50 min), rapid water uptake is observed, followed by a slight decrease in mass for G2, which may result from the partial leaching of soluble components or structural rearrangements within the polymer network. These findings highlight the pronounced effect of the polymer matrix composition and the nature of crosslinking on the water absorption kinetics in acidic media.

Figure 2d illustrates the swelling behavior of the samples in an alkaline medium. Sample G2 shows a rapid increase in swelling followed by stabilization at approximately 0.55 g, whereas G1 exhibits a slower swelling process, reaching around 0.3 g. Samples A3 and A4 display minimal swelling (~0.1–0.15 g), indicating low sensitivity to alkaline conditions. These observations confirm that water uptake is strongly dependent on the composition and structural characteristics of the polysaccharides and emphasize the importance of ionic interactions and crosslinking in controlling the swelling kinetics.

Physically crosslinked gellan gum hydrogels are known to exhibit poor structural stability under physiological conditions. This limitation mainly arises from the gradual substitution of divalent ions with monovalent cations in aqueous media, leading to partial disruption of the polymer network. Such degradation becomes critical when the material is intended for biomedical use, particularly in wound dressings, where it must retain integrity for at least the first two to four weeks of the healing process [45,46].

**Figure 3:**
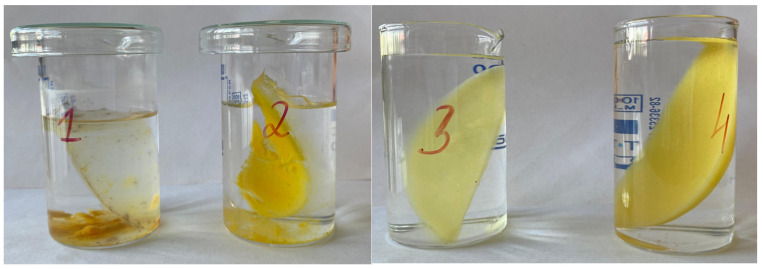
Stability evaluation of hydrogel samples over a 4-month period: gellan gum–based hydrogels (**left**, numbers 1–2) and agar–agar–based hydrogels (**right**, numbers 3–4).

To evaluate the durability of the synthesized samples, their stability in aqueous environments was monitored over four months. Samples 1 and 2 began to delaminate within three to four days after immersion, forming distinct layers that persisted until the end of the experiment (Figure 3). Due to this rapid loss of cohesion, these specimens were not subjected to detailed quantitative testing, since reliable measurement of their swelling and sol–gel parameters was not feasible.

**Figure 4:**
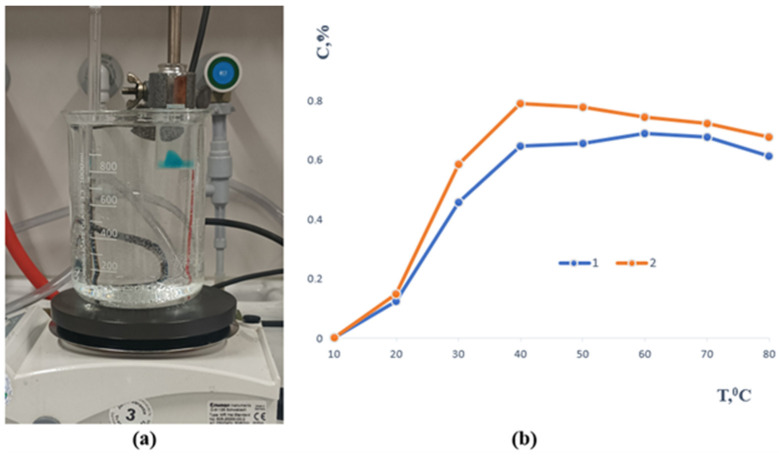
(**a**) Experimental setup illustrating the visual observation of the release process. (**b**) Release of methylene blue from the samples as a function of temperature (1—gellan gum and 2—agar, respectively).

In contrast, samples 3 and 4 remained intact throughout the study, demonstrating excellent long-term stability (Figure 3). The sol–gel fraction analysis showed that before immersion, their values were 1.4046 and 2.0308, respectively, while after seven days of extraction they decreased to 1.1096 and 1.6131. This reduction indicates partial removal of unbound polymer chains while confirming the formation of a sufficiently crosslinked and stable network capable of maintaining its structural integrity over time. All samples underwent mechanical testing after the stability evaluation, with an emphasis on tensile strength. The temperature-dependent release profile of methylene blue from the polymer-based sample containing gellan gum is depicted in Figure 4. A slow release of the encapsulated component was seen when the material was first submerged in an aqueous solution at 20 °C.

As the temperature rose, the release rate increased as well, peaking at about 39 to 40 °C, or conditions that are very similar to physiological hyperthermia. After this, the re-lease pattern for both formulas was comparatively consistent. Complete methylene blue transfer into the aqueous phase was seen at high temperatures of 80 °C. Furthermore, the polymer matrix itself completely dissolved in these circumstances, demonstrating the carrier system’s heat fragility. All of the aforementioned samples will continue to be studied.

**Figure 5:**
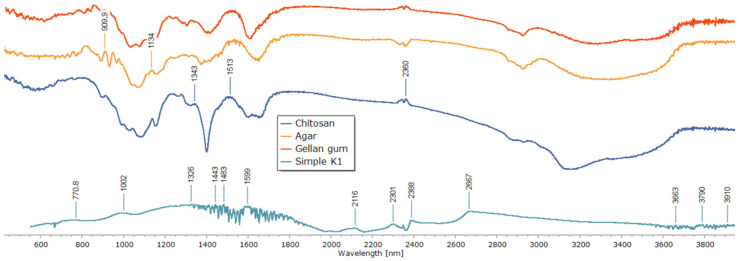
FTIR spectra of individual polysaccharides and the composite patch.

To clarify the chemical interactions and crosslinking mechanism within the polysaccharide matrix, Fourier-transform infrared (FTIR) spectroscopy was used (Figure 5). Individual polymer spectra of agar, gellan gum, and chitosan revealed distinctive absorption bands that matched their functional groups. While the bands at 2850–2920 cm^−1^ correlated with C–H stretching, the broad peaks around 3400–3500 cm^−1^ were ascribed to O–H and N–H stretching vibrations. The chitosan spectra showed a signal associated with C–O–C stretching at about 1020 cm^−1^ and a characteristic band at 1598 cm^−1^ (N–H bending, amide II). The gellan and agar spectra revealed glycosidic bond vibrations close to 1000 cm^−1^ and asymmetric COO^−^ stretching vibrations at 1536 and 1518 cm^−1^.

Significant spectrum alterations were seen in the composite sample (Simple K1) that was crosslinked only with glutaraldehyde. Stronger hydrogen bonding and the consumption of free hydroxyl and amino groups are indicated by the broad O–H/N–H stretching band shifting to lower wavenumbers. The production of imine (C=N) bonds via the Schiff base reaction between chitosan amino groups and glutaraldehyde aldehyde groups was validated by the presence and intensity of the band in the 1501–1598 cm^−1^ region. While the attenuation of the 1000 cm^−1^ band indicated structural change of the polysaccharide backbone, the decreased intensity of the 1326 and 1239 cm^−1^ peaks indicated the involvement of C–O–C and C–N links in crosslinking.

It was confirmed that matrix stabilization happened only through covalent links without the involvement of metal ions because no new bands were found in the low-frequency region (500–600 cm^−1^), which is characteristic for metal–oxygen coordination bonds. All things considered, the detected spectrum alterations offer compelling proof of effective covalent network creation via imine bond production, guaranteeing improved structural stability of the resulting composite.

The resulting hydrogel patches had consistent thickness throughout the surface, with variances of no more than 0.3–0.5%. The polymer network’s consistent gelation and the casting process’s repeatability were confirmed by the observed average thickness values, which varied from 42 to 70 µm. High mechanical integrity of the manufactured films was also shown by the fact that all patches showed good flexibility and folding endurance, withstanding up to 70 folds without obvious cracks or deformation.

These findings are in line with earlier research on chitosan–glutaraldehyde crosslinking systems, where O–H/N–H band shifts and the appearance of C=N vibrations at 1550–1590 cm^−1^ were found to be important markers of the formation of covalent bonds [47,48]. The lack of coordination bands associated with Ca^2+^ further supports the idea that system’s network stabilization was entirely covalent.

**Figure 6:**
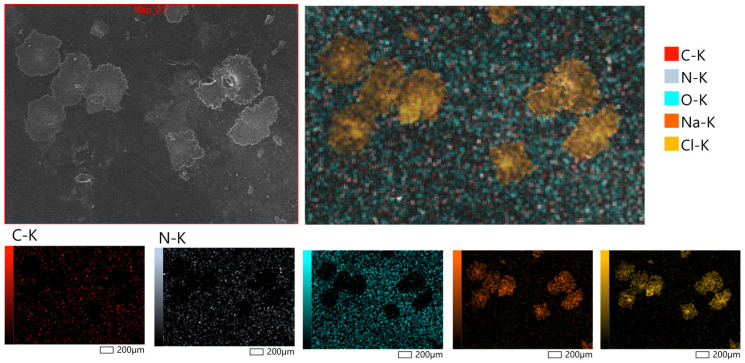
Surface morphology of hydrogel patches at magnifications of 200×, obtained by scanning electron microscopy (SEM, JSM-6390, JEOL Ltd., Tokyo, Japan).

Figure 6 displays representative micrographs taken at magnifications of 200× to examine the surface morphology of the hydrogel patches that were generated. X-ray microanalysis further verified that the hydrogel patches under examination had a rough and porous surface topology. The partial rupture of the polymeric framework during the drying process is responsible for the surface’s uneven fractures. It is anticipated that these morphological characteristics will facilitate water penetration into the polymer network, increasing the material’s capacity to hydrate and promoting moderate swelling behavior. Bao et al. [49] observed similar surface properties of hydrogels based on polysaccharides, confirming the repeatability of these structural features.

## 4. Conclusions

The results demonstrated that glutaraldehyde effectively crosslinked gellan gum, chitosan, and agar–agar within a single polymeric network, producing a stable and flexible hydrogel film. FTIR data confirmed covalent network formation through imine bonding, while SEM analysis visualized a uniform microporous structure supporting moisture retention and diffusion. The composite exhibited pH-dependent swelling (410% at pH 9.18 vs. 275% at pH 4.01), tensile strength of 0.82 MPa, and elongation of 42%, ensuring mechanical reliability under physiological conditions. Methylene blue release increased to 78% after 24 h at 40 °C, confirming diffusion-governed kinetics. Collectively, these findings suggest that the gellan–chitosan–agar hydrogel system crosslinked by glutaraldehyde is a promising candidate for transdermal and wound-healing applications due to its structural integrity, responsiveness, and biocompatibility.

## Figures and Tables

**Table 1:** Composition of formulations with different feed ratios.

Samples	Gellan Gum, (g)	Chitosan, (g)	Agar, (g)	Glutaraldehyde	Methylene Blue, (g)
G1	1	0.25	-	2.5	-
G2	1	0.5	-	2.5	-
A1	-	0.25	1	2.5	-
A2	-	0.5	1	2.5	-
C5	0.5	0.25	0.5	2.5	-
C6	0.5	0.125	0.5	2.5	-
GM	1	0.25	-	2.5	0.01
AM	-	0.25	1	2.5	0.01

**Table 2:** Weight variation, thickness and folding endurance of prepared patches.

Formulation Code	Weight Variation (g)	Mean Thickness (mm)	Folding Endurance
G1	5.90 ± 1.03	0.2175	42 ± 0.5
G2	6.47 ± 1.07	0.2545	51 ± 0.6
A1	5.43 ± 0.96	0.1195	63 ± 0.7
A2	5.87 ± 0.84	0.1236	70 ± 0.3

## Data Availability

The datasets used and/or analyzed during the present study are available from the corresponding author on reasonable request.

## References

[1] Bharadwaj S., Garg V.K., Sharma P.K., Bansal M., Kumar N. (2011). Recent Advancement in Transdermal Drug Delivery System. Int. J. Pharma Prof. Res..

[2] Dev M.J., Warke R.G., Warke G.M., Mahajan G.B., Patil T.A., Singhal R.S. (2022). Advances in Fermentative Production, Purification, Characterization, and Applications of Gellan Gum. Bioresour. Technol..

[3] Lalebeigi F., Alimohamadi A., Afarin S., Aghamirza Moghim Aliabadi H., Mahdavi M., Farahbakhshpour F., Hashemiaval N., Kalantari Khandani K., Eivazzadeh-Keihan R., Maleki A. (2024). Recent Advances on Biomedical Applications of Gellan Gum: A Review. Carbohydr. Polym..

[4] Shukla R., Kashaw S.K., Jain A.P., Lodhi S. (2016). Fabrication of Apigenin Loaded Gellan Gum–Chitosan Hydrogels (GGCH-HGs) for Effective Diabetic Wound Healing. Int. J. Biol. Macromol..

[5] Reczyńska-Kolman K., Hartman K., Kwiecień K., Brzychczy-Włoch M., Pamuła E. (2021). Composites Based on Gellan Gum, Alginate and Nisin-Enriched Lipid Nanoparticles for the Treatment of Infected Wounds. Int. J. Mol. Sci..

[6] Zia K.M., Tabasum S., Khan M.F., Akram N., Akhter N., Noreen A., Zuber M. (2018). Recent Trends on Gellan Gum Blends with Natural and Synthetic Polymers: A Review. Int. J. Biol. Macromol..

[7] Gering C., Rasheed A., Koivisto J.T., Párraga J., Tuukkanen S., Kellomäki M. (2021). Chemical Modification Strategies for Viscosity-Dependent Processing of Gellan Gum. Carbohydr. Polym..

[8] Xu Z., Li Z., Jiang S., Bratlie K.M. (2018). Chemically Modified Gellan Gum Hydrogels with Tunable Properties for Use as Tissue Engineering Scaffolds. ACS Omega.

[9] Krauland A.H., Leitner V.M., Bernkop—Schnürch A. (2003). Improvement in the in Situ Gelling Properties of Deacetylated Gellan Gum by the Immobilization of Thiol Groups. J. Pharm. Sci..

[10] Pańczyszyn E., Jaśko M., Miłek O., Niedziela M., Męcik-Kronenberg T., Hoang-Bujnowicz A., Zięba M., Adamus G., Kowalczuk M., Osyczka A.M. (2021). Gellan Gum Hydrogels Cross-Linked with Carbodiimide Stimulates Vacuolation of Human Tooth-Derived Stem Cells in Vitro. Toxicol. In Vitro.

[11] Cencetti C., Bellini D., Longinotti C., Martinelli A., Matricardi P. (2011). Preparation and Characterization of a New Gellan Gum and Sulphated Hyaluronic Acid Hydrogel Designed for Epidural Scar Prevention. J. Mater. Sci. Mater. Med..

[12] Zhang J., Tan W., Li Q., Liu X., Guo Z. (2021). Preparation of Cross-Linked Chitosan Quaternary Ammonium Salt Hydrogel Films Loading Drug of Gentamicin Sulfate for Antibacterial Wound Dressing. Mar. Drugs.

[13] Nair S.S. (2019). Chitosan-Based Transdermal Drug Delivery Systems to Overcome Skin Barrier Functions. J. Drug Deliv. Ther..

[14] Grabska-Zielińska S. (2024). Cross-Linking Agents in Three-Component Materials Dedicated to Biomedical Applications: A Review. Polymers.

[15] Jayakumar R., Prabaharan M., Nair S.V., Tamura H. (2011). Biomaterials Based on Chitin and Chitosan for Wound Dressing Applications. Biotechnol. Adv..

[16] Dhivya S., Padma V.V., Santhini E. (2015). Wound Dressings—A Review. BioMed.

[17] Ravi Kumar M.N.V. (2000). A Review of Chitin and Chitosan Applications. React. Funct. Polym..

[18] Shah J., Patel D., Rananavare D., Hudson D., Tran M., Schloss R., Langrana N., Berthiaume F., Kumar S. (2025). Recent Advancements in Chitosan-Based Biomaterials for Wound Healing. J. Funct. Biomater..

[19] Kim I.Y., Seo S.J., Moon H.S., Yoo M.K., Park I.Y., Kim B.C., Cho C.S. (2008). Chitosan and Its Derivatives for Tissue Engineering Applications. Biotechnol. Adv..

[20] Zhao X., Wu H., Guo B., Dong R., Qiu Y., Ma P.X. (2017). Antibacterial Anti-Oxidant Electroactive Injectable Hydrogel as Self-Healing Wound Dressing with Hemostasis and Adhesiveness for Cutaneous Wound Healing. Biomaterials.

[21] El-Gendy N., Abdelbary G., EL-Komy M., Saafan A. (2009). Design and Evaluation of a Bioadhesive Patch for Topical Delivery of Gentamicin Sulphate. Curr. Drug Deliv..

[22] Takeuchi Y., Ikeda N., Tahara K., Takeuchi H. (2020). Mechanical Characteristics of Orally Disintegrating Films: Comparison of Folding Endurance and Tensile Properties. Int. J. Pharm..

[23] Alven S., Peter S., Aderibigbe B.A. (2022). Polymer-Based Hydrogels Enriched with Essential Oils: A Promising Approach for the Treatment of Infected Wounds. Polymers.

[24] Che X., Zhao T., Hu J., Yang K., Ma N., Li A., Sun Q., Ding C., Ding Q. (2024). Application of Chitosan-Based Hydrogel in Promoting Wound Healing: A Review. Polymers.

[25] Vatanpour V., Yavuzturk Gul B., Zeytuncu B., Korkut S., İlyasoğlu G., Turken T., Badawi M., Koyuncu I., Saeb M.R. (2022). Polysaccharides in Fabrication of Membranes: A Review. Carbohydr. Polym..

[26] Abdollahi H., Amiri S., Amiri F., Moradi S., Zarrintaj P. (2024). Antibacterial Biocomposite Based on Chitosan/Pluronic/Agarose Noncovalent Hydrogel: Controlled Drug Delivery by Alginate/Tetracycline Beads System. J. Funct. Biomater..

[27] Craciun A.M., Morariu S., Marin L. (2022). Self-Healing Chitosan Hydrogels: Preparation and Rheological Characterization. Polymers.

[28] Berradi A., Aziz F., Achaby M.E., Ouazzani N., Mandi L. (2023). A Comprehensive Review of Polysaccharide-Based Hydrogels as Promising Biomaterials. Polymers.

[29] Zawawi N.A., Maarof M., Fadilah N.I.M., Hao D.L.Q., Tabata Y., Fauzi M.B. (2025). Hybrid Adhesive Hydrogel Patch Containing Genipin-Crosslinked Gelatin–Hyaluronic Acid for Future Use in Atopic Dermatitis. J. Funct. Biomater..

[30] Tatykhanova G., Aseyev V., Vamvakaki M., Khutoryanskiy V., Kudaibergenov S. (2022). Ophthalmic drug delivery system based on the complex of gellan and ofloxacin. KazNU Chem. Bull..

[31] Collinson M.M. (1999). Sol-Gel Strategies for the Preparation of Selective Materials for Chemical Analysis. Crit. Rev. Anal. Chem..

[32] Rajawasam C.W.H., Dodo O.J., Weerasinghe M.A.S.N., Raji I.O., Wanasinghe S.V., Konkolewicz D., De Alwis Watuthanthrige N. (2024). Educational Series: Characterizing Crosslinked Polymer Networks. Polym. Chem..

[33] Karadag E. (1996). In Vitro Swelling Studies and Preliminary Biocompatibility Evaluation of Acrylamide-Based Hydrogels. Biomaterials.

[34] Arshad J., Barkat K., Ashraf M.U., Badshah S.F., Ahmad Z., Anjum I., Shabbir M., Mehmood Y., Khalid I., Malik N.S. (2023). Preparation and Characterization of Polymeric Cross-Linked Hydrogel Patch for Topical Delivery of Gentamicin. e-Polymers.

[35] Ahmad S., Usman Minhas M., Ahmad M., Sohail M., Abdullah O., Khan K.U. (2018). Topical Hydrogel Patches of Vinyl Monomers Containing Mupirocin for Skin Injuries: Synthesis and Evaluation. Adv. Polym. Technol..

[36] Nokoorani Y.D., Shamloo A., Bahadoran M., Moravvej H. (2021). Fabrication and Characterization of Scaffolds Containing Different Amounts of Allantoin for Skin Tissue Engineering. Sci. Rep..

[37] Bakhrushina E.O., Shumkova M.M., Avdonina Y.V., Ananian A.A., Babazadeh M., Pouya G., Grikh V.V., Zubareva I.M., Kosenkova S.I., Krasnyuk I.I. (2025). Transdermal Drug Delivery Systems: Methods for Enhancing Skin Permeability and Their Evaluation. Pharmaceutics.

[38] Jeong W.Y., Kwon M., Choi H.E., Kim K.S. (2021). Recent Advances in Transdermal Drug Delivery Systems: A Review. Biomater. Res..

[39] Sivadasan D., Madkhali O.A. (2024). The Design Features, Quality by Design Approach, Characterization, Therapeutic Applications, and Clinical Considerations of Transdermal Drug Delivery Systems—A Comprehensive Review. Pharmaceuticals.

[40] Patel D., Chaudhary S.A., Parmar B., Bhura N. (2012). Transdermal Drug Delivery System: A Review Article. PharmaTutor.

[41] Malvey S., Rao J.V., Arumugam K.M. (2019). Transdermal Drug Delivery System: A Mini Review. Pharma Innov. J..

[42] Feketshane Z., Alven S., Aderibigbe B.A. (2022). Gellan Gum in Wound Dressing Scaffolds. Polymers.

[43] European Medicines Agency (EMA) (2014). Guideline on the Quality of Transdermal Patches (EMA/CHMP/QWP/608924/2014).

[44] Al Hanbali O.A., Khan H.M.S., Sarfraz M., Arafat M., Ijaz S., Hameed A. (2019). Transdermal Patches: Design and Current Approaches to Painless Drug Delivery. Acta Pharm..

[45] Gao L., Gan H., Meng Z., Gu R., Wu Z., Zhu X., Sun W., Li J., Zheng Y., Sun T. (2016). Evaluation of Genipin-Crosslinked Chitosan Hydrogels as a Potential Carrier for Silver Sulfadiazine Nanocrystals. Colloids Surf. B Biointerfaces.

[46] Ibrahim M.M., Nair A.B., Aldhubiab B.E., Shehata T.M., Catala A. (2017). Hydrogels and Their Combination with Liposomes, Niosomes, or Transfersomes for Dermal and Transdermal Drug Delivery. Liposomes.

[47] Udoetok I.A., Karoyo A.H., Mohamed M.H., Wilson L.D. (2024). Chitosan Biocomposites with Variable Cross-Linking and Copper-Doping for Enhanced Phosphate Removal. Molecules.

[48] Cheng Y., Mondal A.K., Wu S., Xu D., Ning D., Ni Y., Huang F. (2020). Study on the Anti-Biodegradation Property of Tunicate Cellulose. Polymers.

[49] Bao Y., Ma J., Li N. (2011). Synthesis and Swelling Behaviors of Sodium Carboxymethyl Cellulose-g-Poly(AA-Co-AM-Co-AMPS)/MMT Superabsorbent Hydrogel. Carbohydr. Polym..

